# Explainability and human intervention in autonomous scanning probe microscopy

**DOI:** 10.1016/j.patter.2023.100858

**Published:** 2023-10-09

**Authors:** Yongtao Liu, Maxim A. Ziatdinov, Rama K. Vasudevan, Sergei V. Kalinin

**Affiliations:** 1 Center for Nanophase Materials Sciences, Oak Ridge National Laboratory, Oak Ridge, TN 37830, USA; 2 Computational Sciences and Engineering Division, Oak Ridge National Laboratory, Oak Ridge, TN 37830, USA; 3 Department of Materials Science and Engineering, University of Tennessee, Knoxville, TN 37996, USA

**Keywords:** autonomous experiments, human in the loop, Gaussian process, deep kernel learning, scanning probe microscopy

## Abstract

The broad adoption of machine learning (ML)-based autonomous experiments (AEs) in material characterization and synthesis requires strategies development for understanding and intervention in the experimental workflow. Here, we introduce and realize a post-experimental analysis strategy for deep kernel learning-based autonomous scanning probe microscopy. This approach yields real-time and post-experimental indicators for the progression of an active learning process interacting with an experimental system. We further illustrate how this approach can be applied to human-in-the-loop AEs, where human operators make high-level decisions at high latencies setting the policies for AEs, and the ML algorithm performs low-level, fast decisions. The proposed approach is universal and can be extended to other techniques and applications such as combinatorial library analysis.

## Highlights

• Active learning-driven experiments of autonomous nature accelerate scientific discovery
• A post-experimental analysis approach for autonomous experiments is developed
• The approach provides real-time and post-experimental insights
• The approach can be extended to human-in-the-loop autonomous experiments

## The bigger picture

Machine learning-empowered autonomous experiments are transforming the future of scientific research and discovery. We developed a post-experimental analysis approach based on deep kernel active learning-driven autonomous scanning probe microscopy workflow that can be utilized in any physical imaging automated experimental platform with various imaging spectroscopic methods. This approach offers automated experiment interpretation and the ability to extend to human interventions in automated experiments. Human operators make high-level decisions while machine learning analyzes data and makes low-level decisions, providing a real-time combination of expertise and efficiency in conducting experiments.

## Graphical abstract

**Figure:**
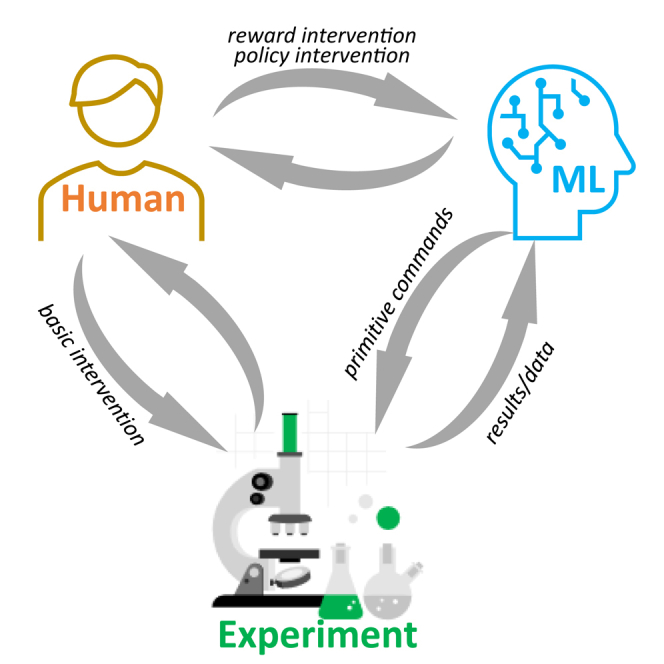

## Teaser

This proposed approach provides real-time and post-experimental insights into decision-making processes in machine learning-driven autonomous experiments, which can also be applied to human-in-the-loop alternatives. By combining human expertise and the power of machine learning, this approach can accelerate scientific discovery.

## Introduction

Over the last several years, the materials science community has been firmly riveted by the introduction and optimization of automated experiments in the areas of material synthesis and characterization. For materials synthesis, multiple approaches including pipetting robots,^1^^,^^2^ self-driving labs,^3^^,^^4^^,^^5^ and high-throughput synthesis workflows have been proposed.^6^^,^^7^^,^^8^^,^^9^^,^^10^^,^^11^ For materials characterization, several groups have been developing automated and autonomous experiment (AE) approaches in areas including scanning transmission electron microscopy (STEM),^12^^,^^13^^,^^14^^,^^15^ scanning probe microscopy (SPM),^16^^,^^17^^,^^18^^,^^19^^,^^20^^,^^21^^,^^22^^,^^23^^,^^24^^,^^25^ neutron diffraction,^26^^,^^27^ and X-ray scattering.^28^

The central concept in AE is the workflow,^29^^,^^30^ defined as the sequence of steps and operations performed by automated laboratories or measurement tools. Generally, a workflow can combine steps performed by human and non-human agents. For example, for many microscope operations (e.g., tuning microscope parameters) can be performed automatically, but specific decisions for physical discoveries (e.g., selection of measurement regions) are currently performed by human operators. Similarly, in materials synthesis, the optimization of the specific synthesis conditions can be performed via an automated synthesis platform, but the selection of the endmembers for determining materials’ properties is often manual.

Until now, most of these automated efforts have been based on human-designed workflows, with the role of any machine learning (ML) algorithm limited to (often greedy) optimization of the consecutive process steps. In this process, each step of the workflow is optimized individually, whereas possible correlations between individual steps are ignored. The recent advances in approaches such as deep kernel active learning (DKL) are enabling the development of single-step, beyond-human workflows, as exemplified by the property discovery in scanning probe^20^^,^^23^^,^^31^ and electron microscopy.^14^

The broad deployment of both human-based and non-human workflows for AE necessitates the development of a methodology to monitor the progression of active learning processes interacting with experimental physical systems and to tune the AE progression on a timescale affordable for humans. This includes both developing tools for the explainability of the AE in real time and post experiment, as well as the creation of frameworks that allow human intervention on timescales and decision-making levels amenable to human scientists.

Here we introduce the concept of post-experimental analysis of AE and human-in-the-loop interventions. We demonstrate this approach for SPM with DKL, but this concept is equally applicable for STEM and other Gaussian process-driven materials synthesis and characterization in automated labs and theory exploration over large chemical spaces.^10^^,^^32^^,^^33^^,^^34^^,^^35^

## Results

We consider the general process of post-experimental analysis for AE and introduce key concepts necessary in this case. The central element of experimental active learning is the ML agent iteratively interacting with the experimental system, both performing the experimentation and updating the state of the ML agent. Initially, the ML agent’s state is defined by priors and inferential biases (e.g., a hypothesis list, invariances, pre-trained networks) formed based on human input. Throughout the experiment, the state of the agent is updated in response to the incoming information from the active data generation process (i.e., microscope). Based on the current state and prior information, the agent makes decisions that are communicated to the microscope. This iterative cycle continues until the experimental budget is exhausted or the predefined goal is achieved. At the end of the experiment, the change in the state of the agent represents the knowledge gained during the experiment. Correspondingly, we define the AE post-experimental analysis as the analysis of the decision-making at each experiment step, comparing the decisions made by the agent in the real-time state and the decisions made by the fully trained agent. It is also important to note that the AE post-experimental analysis assessment can include a human component; i.e., based on the real-time experimental results and the AE progression, the human operator can choose to change the nature of the information available to the agent or policies that guide the decision-making and explore the experimental path.

Here, we discuss the AE post-experimental analysis for a specific case of a DKL AE workflow for SPM. Traditionally, SPM is operated by human operators with a certain small number of stages amenable to automation. A typical SPM imaging workflow starts with sample selection, sample preparation and loading, and microscope tuning. With the sample loaded and microscope initiated, a human operator initiates overview and spectroscopy scans, making subsequent decisions based on results at each step. As a consequence of numerous operations performed by human operators manually, the experiment is a laborious and time-intensive process. Furthermore, the decision-making process by human operators is often biased depending on human interest, intention, knowledge, etc., and it is often slow because it is challenging for a human to understand high-dimensional datasets and their relationship in a short time frame. In contrast, ML algorithms can analyze high-dimensional datasets quickly; e.g., deep learning can learn a relationship between thousands of structural image patches and spectroscopic properties. Bayesian methods^36^^,^^37^ exemplified DKL by allowing an active learning process,^31^ making decisions based on past acquired information. When implementing a workflow with DKL in an operating SPM,^20^^,^^23^^,^^31^^,^^38^ the microscope can perform the measurement, process data, make decisions to move the probe, and initiate image scan and/or spectra measurement automatically without human intervention. This largely surpasses the speed of measurements carried out by human operators, accelerating physics discovery. However, tuning this process requires understanding the decisions made by the ML agent and adjusting the policies that guide these decisions. Here we discuss the DKL process and the AE post-experimental analysis. The DKL analysis is based on a pre-acquired dataset with known ground truth; here, using a pre-acquired dataset allows us to compare different experimental paths. However, the methodologies developed here can be straightforwardly implemented on active microscopes. We also note that the same logic can be applied to other experiments as well, e.g., molecular design, processing trajectory optimization, etc.

**Figure 1:**
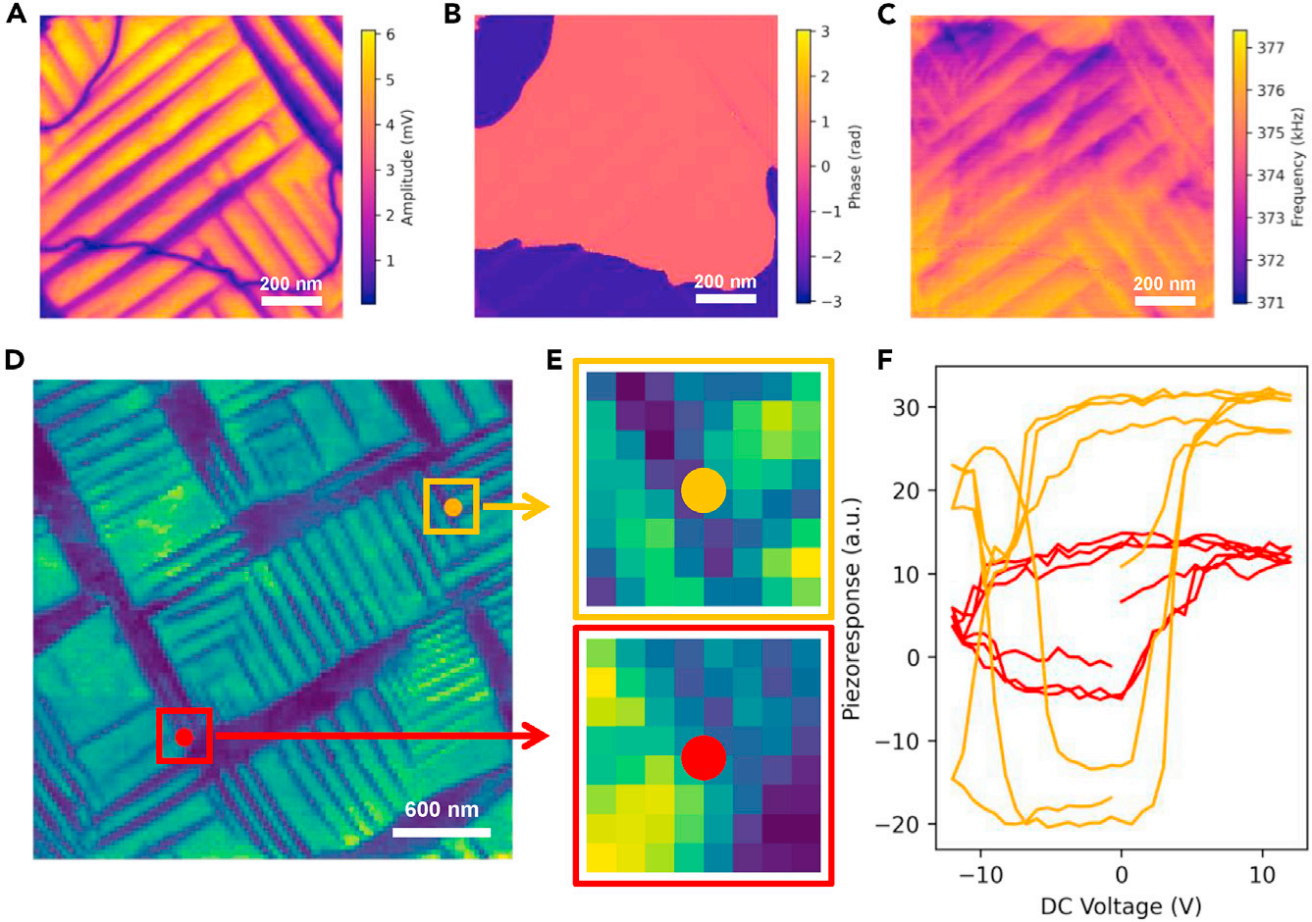
Band excitation piezoresponse force microscopy (BEPFM) and spectroscopy (BEPS) results of model sample PbTiO_3_ (A–C) BEPFM amplitude, phase, and frequency images showing ferroelectric and ferroelastic domain structures in PTO thin film. (D–F) BEPS results of PTO thin film. (E) and (F) show two example domain structures and corresponding spectroscopic behaviors. Note that the structural image patch is sampled throughout the whole image in (D). (D)–(F) are reproduced with permission from Liu et al.^31^

We have chosen band excitation piezoresponse spectroscopy (BEPS) data of a PbTiO_3_ (PTO) thin film as model ground truth data.^31^ The PTO thin film was grown on a (001) KTaO_3_ substrate with a SrRuO_3_ conducting layer. The band excitation piezoresponse force microscopy^39^ (BEPFM) imaging of this film is shown in Figures 1A–1C. The black domains in Figure 1A amplitude image are *a* domains with in-plane polarization, and the bright domains are *c* domains with out-of-plane polarization, indicating the presence of typical *a-c* domain structures in this PTO film. The dark and bright domains in Figure 1B phase image show the antiparallel *c*^+^ and *c*^−^ out-of-plane polarized domains. In addition, the resonance frequency image in Figure 1C also shows the ferroelastic *a-c* domains. In our earlier work, we revealed a mutual interaction among the image channels in BEPFM via causal physical mechanism analysis.^40^ We also implemented DKL in operating SPM for AEs to explore the structure-property relationship in this sample,^23^^,^^31^ and we discovered the relationship between ferroelectric domain structure and polarization-voltage hysteresis loop.

Here, we use this grid BEPS data acquired in our previous work^31^ as a model to illustrate DKL analyses and to establish an AE post-experimental analysis workflow: these grid spectroscopy data provide us with a low-resolution image showing domain structure and corresponding spectroscopy of polarization-voltage hysteresis at each pixel. The BEPS data are shown in Figures 1D–1F. Figure 1D is the image showing the ferroelectric domain structure, Figure 1E shows two example patches that will be used as structural data in DKL analyses, and Figure 1F shows two example polarization-voltage hystereses corresponding to these patches.

**Figure 2:**
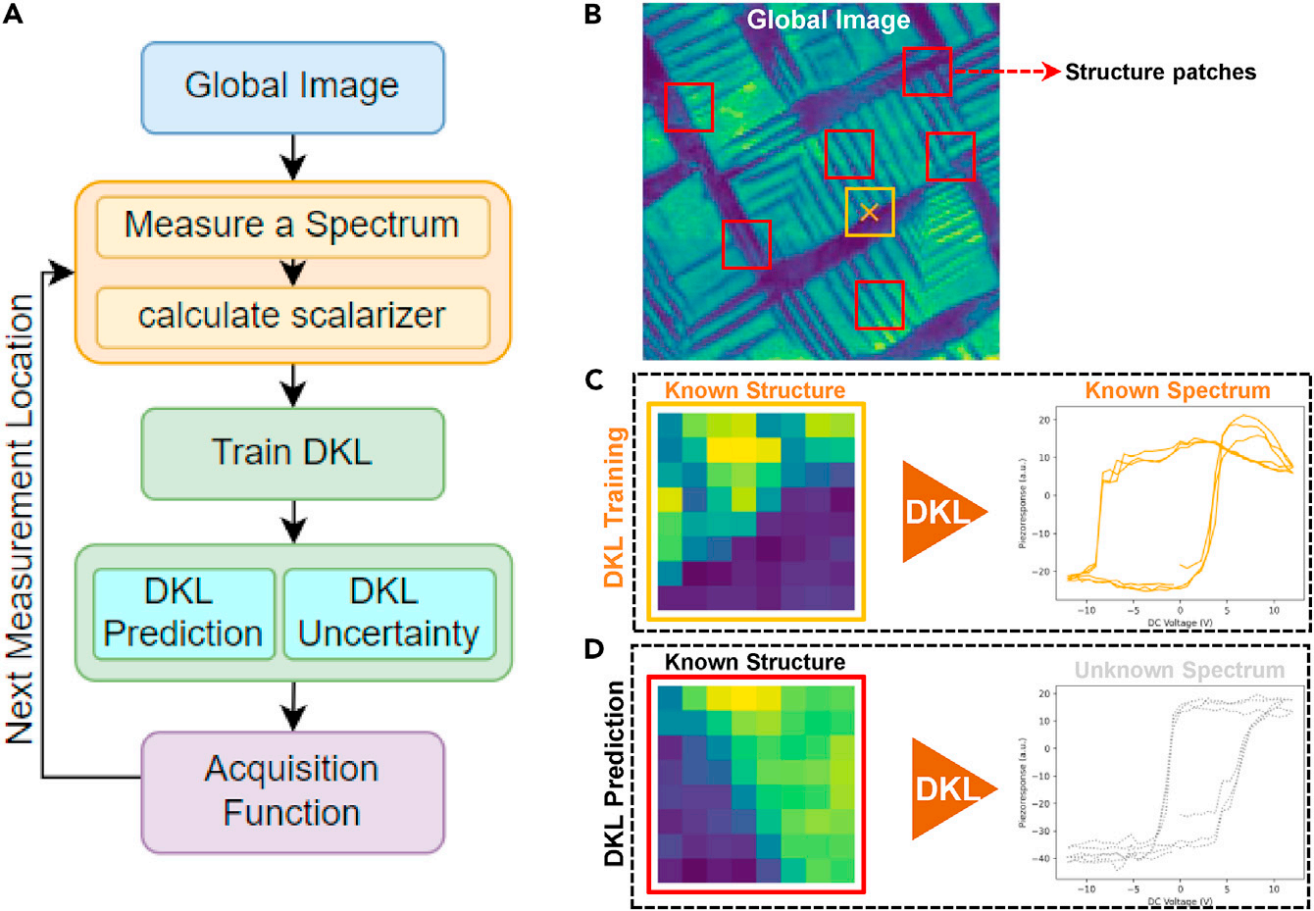
DKL workflow (A) Workflow of DKL-driven SPM measurement. (B) Global structural image. (C) DKL training with known structure and known spectrum. (D) DKL prediction with known structure; DKL predicts the values of scalarizer function (i.e., characteristic of the spectrum that is of interest to experimentalists, such as the area under hysteresis loop, magnitude of the signal at a certain voltage, etc.).

In the DKL experiment shown in Figure 2, the agent has access to the global image (e.g., Figure 2B). The global image can be sampled at the individual location [$x_{i}$, $y_{i}$] to return the local image patch showing known structure (e.g., squared regions in Figure 2B) and allows for local spectroscopy measurement (e.g., the spectrum shown in Figure 2C) at the same location. The (image patch, spectrum) pair represents the feature (input) and target (output) of the DKL process, where at the initial stage, all the features and a small number of targets are available. During the active learning process, the algorithm (1) identifies the next feature (i.e., location [$x_{i+1}$, $y_{i+1}$]) to measure, (2) provides the coordinates to the microscope, (3) receives new data and appends it to the target list, and (4) updates the model with the expanded list of targets. The general task of the exploratory DKL algorithm is to learn the relationship between features and targets by iteratively selecting the next sampling location and attempting to arrive at this in the smallest number of steps. For a ferroelectric material, this can be learning the relationship between the local domain structure (patch) and local hysteresis loop (spectrum).

To arrive at the structure-property relationships in the smallest number of steps, the DKL method is used as a basis for a Bayesian optimization (BO)^41^ process. In the DKL BO framework, the chosen characteristic of the spectrum or evolution of spectrum sequence (mutual entropy, etc.) defines the reward function for a DKL learning process. The goal in AE is to maximize this function. For example, DKL can be used to discover the microstructural elements that correspond to the largest area of hysteresis loops,^31^ regions with the highest intensity of edge plasmons in the STEM-electron energy loss spectroscopy (EELS) measurements,^14^ or the highest internal electric field in the 4D STEM.^42^ It is also important to note that DKL AE is an example of beyond-human workflows; the correlation between the spectra and structures is learned in real-time experiments.

The balance between the exploration and exploitation of the BO framework is set via the choice of the acquisition function built upon the “scalarizer” function. We implemented and investigated three acquisition functions in this work, namely expected improvement (EI), upper confidence bound (UCB), and prediction maximum uncertainty (MU). Specifically, for the DKL-PFM experiment, the DKL is trained by image patches ${IP}_{i}$ and measurements $M_{i}$ (chosen as scalarizer function applied to spectrum) at locations [$x_{i}$, $y_{i}$]; the trained DKL predicts unmeasured locations with known structural image patches. The scalarizer function can be the area of the hysteresis loop or the offset of the loop in the voltage axis or any other characteristics of the spectrum. Then, the acquisition function derives the next measurement location [$x_{i+1}$, $y_{i+1}$] based on DKL prediction and uncertainty. We define the sequence of spectroscopy measurement locations (with corresponding image patches) as the *experimental trace*. It is important to note that the decision-making in the DKL AE is based on a single scalar characteristic of the measured spectra (i.e., scalarizer) rather than the full spectrum. However, the availability of the full spectrum allows us to incorporate counterfactual analyses, which will be illustrated below.

Here we introduce the AE post-experimental analysis framework for DKL-BEPS experiment, comprising (1) regret analysis including the acquisition function component analysis and counterfactual decision-making, (2) trajectory analysis and feature discovery, and (3) global latent trajectory analysis.

**Figure 3:**
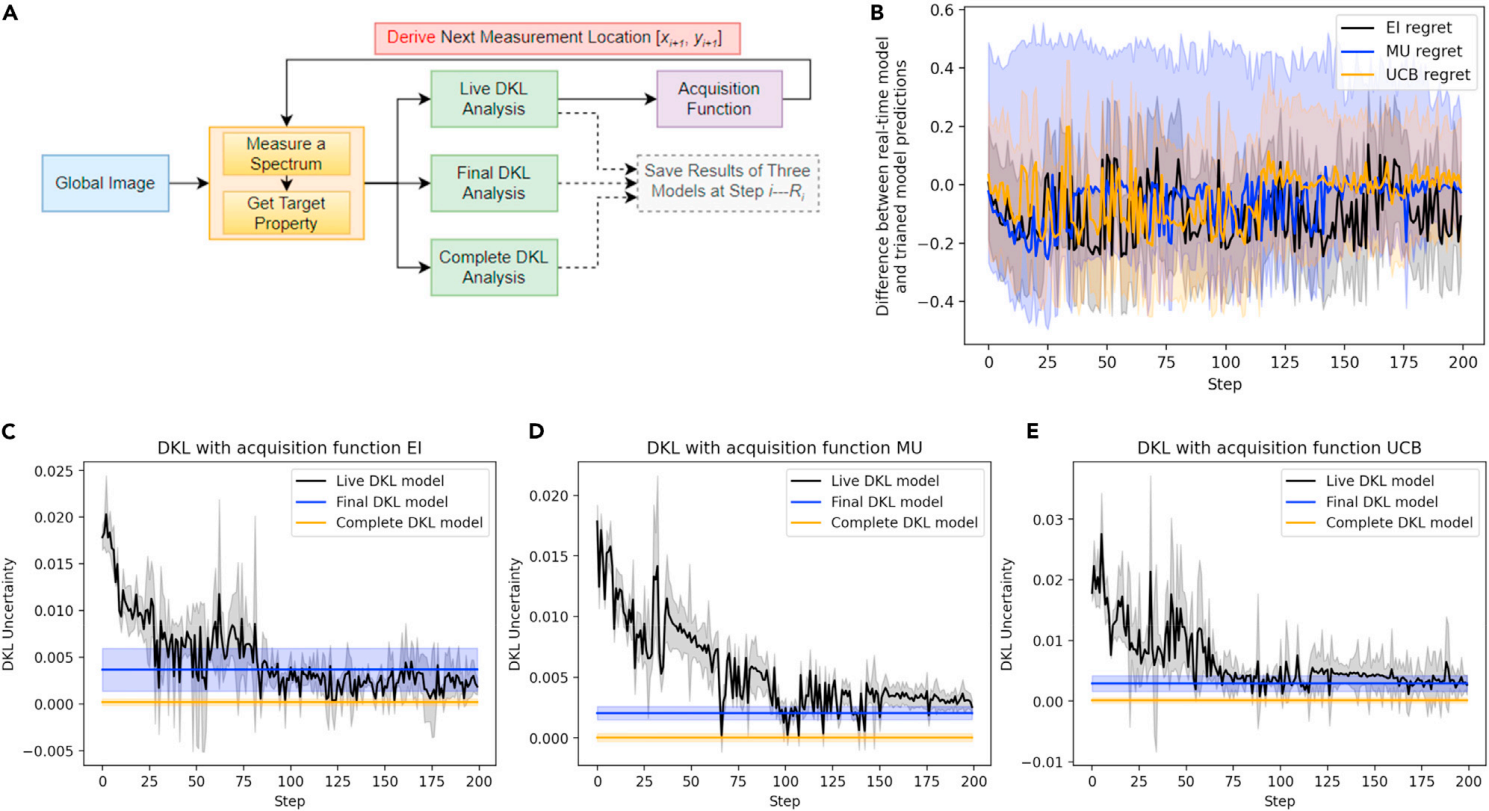
Regret analysis of DKL AE (A) The workflow of regret analysis, where the final DKL and complete DKL models are reference models. The final DKL model is the DKL model after 200 steps of exploration, and the complete DKL model is trained with all available data. (B) Regret of DKL with different acquisition functions. The regret is defined as ${Rg}_{i}=P_{i}^{real-time\;DKL}-P_{i}^{trained\;DKL}$, where the solid line is the mean of *Rg*_*i*_, and the distribution is the deviation of Rg_i_ at step *i*. (C, D, and E) Comparison of live, final, and complete DKL prediction uncertainty as a function of step, (C), (D), and (E) show results of DKL with three different acquisition functions, respectively; here, the solid line shows the average uncertainty, and the shadow shows the deviation of uncertainty.

In regret analysis, we compare the knowledge gain following the experimental traces of the actual AE DKL model and the trained DKL models. To illustrate this concept, we have defined three DKL models as shown in Figure 3. The *live DKL model* is the active model during the DKL experiment, which is trained and updated at each exploration step. This model has learned only from the experimental data available from the beginning of the experiment to the current step. The *final DKL model* is the model trained on all data sampled during the DKL experiment, e.g., in a 200-step DKL experiment, and the final DKL model is trained on 200 sampled image patches and corresponding spectroscopic properties. The *full DKL model* is trained by all data in the model BEPS data. Note that the full DKL model can be trained only when the ground truth data are available, which often is not possible in a real experiment, whereas the active learning experiment allows access to live and (in the end) trained DKL models.

During the post-experimental regret analysis, the predictions of the final DKL and complete DKL models are compared to the prediction of the live DKL model. The regret is defined as the difference between the predicted scalarizer of the live DKL model and the final DKL model:

$${Regret}_{i}={Prediction}_{i}^{live\;DKL}-{Prediction}_{i}^{final\;DKL}$$

The regret evolution for three acquisition functions is shown in Figure 3B. Here, the solid line in Figure 3B indicates the mean ${Regret}_{i}$, and the shadow indicates the deviation of ${Regret}_{i}$ across the dataset (i.e., the standard deviation of the DKL uncertainty image).

The learning process of live DKL models with different acquisition functions is shown in Figures 3C–3E. The DKL prediction uncertainty is used as a metrics of learning here. The uncertainty of the final DKL model and the complete DKL model are shown as a comparison. Here, the prediction uncertainty indicates how well the model can predict the scalarizer value from the structural domain pattern. The black line illustrates the evolution of the uncertainty during AE, whereas the shaded region is the distribution of uncertainties within the image. For the UCB and EI acquisition functions, the model learns faster. However, for EI, there is a clear inflection point suggesting the discovery of a new type of behavior. Experimentally, a smoother learning process can be achieved by increasing the exploratory component of the chosen acquisition function. Curiously, the variability of the predicted behaviors is small at the beginning of the learning process, grows at the intermediate steps when the model discovers new classes of behaviors, and starts to decrease for the second half of the training. The regret function (comparison of the final and live model) for this dataset shows a high noise level and allows us to identify the stage of maximal learning. Finally, the mean prediction component can further be evaluated to characterize the behavior of the optimization part of the algorithm (not shown).

We note that the *learning curves* in Figures 3C–3E can be evaluated during the experiment and hence represent the indicators based on which hyperparameter tuning (choice of acquisition function, tuning the exploration and exploitation within acquisition function, addition of random exploration, e.g., via epsilon-greedy policies) can be introduced.

**Figure 4:**
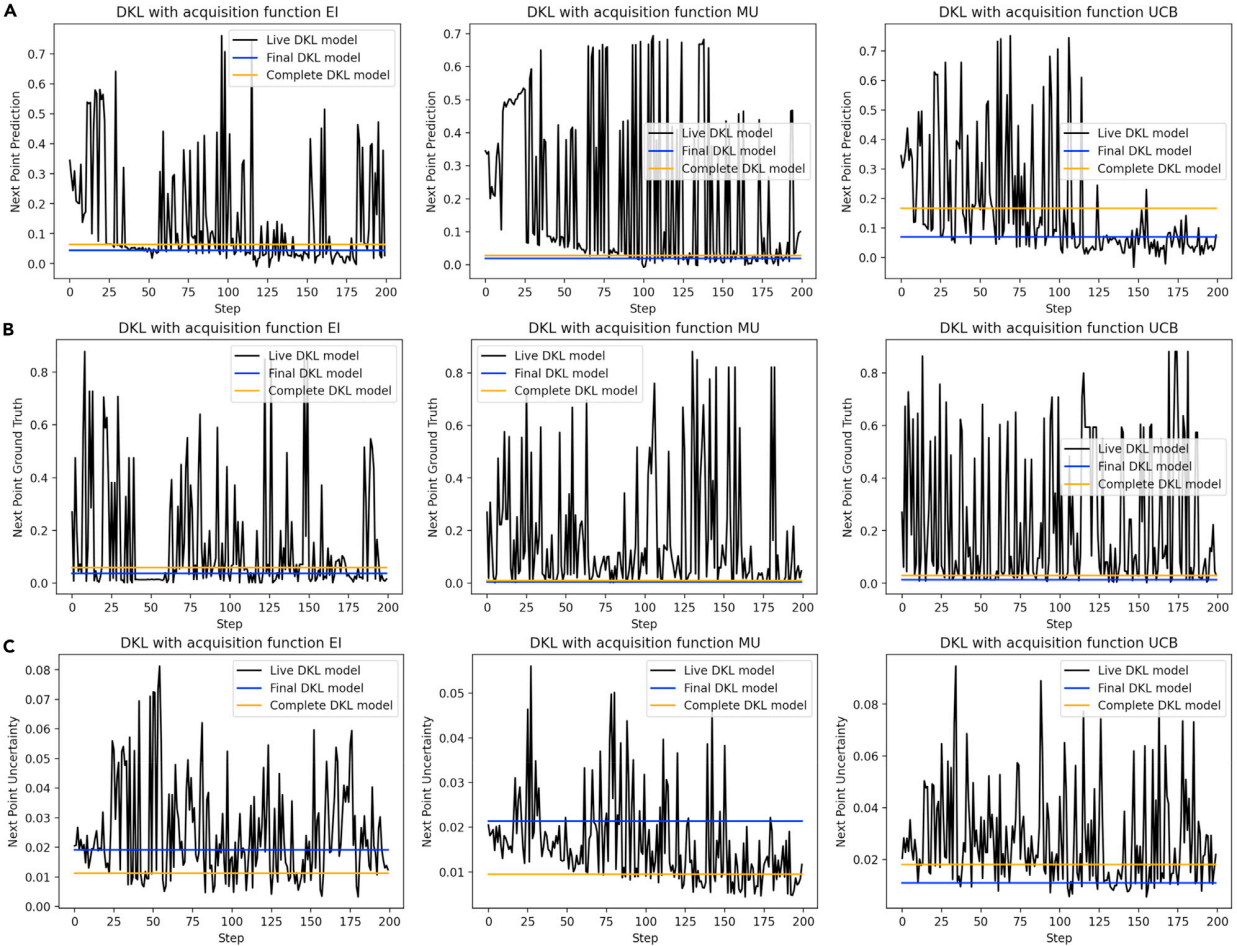
Comparison of live, final, and complete DKL prediction and uncertainty for the next measurement location (A) Shown is the DKL prediction of the value of the next measurement location. (B) Shown is the ground truth of the predicted next point. (C) Shown is the uncertainty of the next point.

A deeper insight into the decision-making process can be given based on the analysis of the components of the acquisition function, including predicted value and uncertainty. Therefore, the DKL predicted value and uncertainty of the next measurement point are shown in Figure 4 as a function of step. The ground truth of the next measurement point is also shown in Figure 4 as a comparison. The observed traces show very high noise levels common for active learning tasks. Note that this high noise level is present both in the prediction and the ground truth data. However, the background trend of the decreasing predictive value (i.e., BO minimizes the scalarizer function) is seen, and the rate of learning for different acquisition functions can be deduced as the bottom envelope of observed behaviors.

Another component of AE post-experimental analysis is counterfactual analysis. The counterfactual analysis is defined as how the action at each step changes if the scalarizer had been chosen to be different. We recall that the progression of the DKL is driven by the chosen characteristic of the spectra defined via the scalarizer function (e.g., the scalarizer can be the switching bias, nucleation bias, and loop area for hysteresis loops in BEPS), and DKL prediction and uncertainty of the scalarizer form the acquisition function and guide the exploration. The reason the counterfactual analysis is possible at each step is that the actual experiment collects the full-spectrum data, and any scalarizer characteristics can be evaluated at each step.

**Figure 5:**
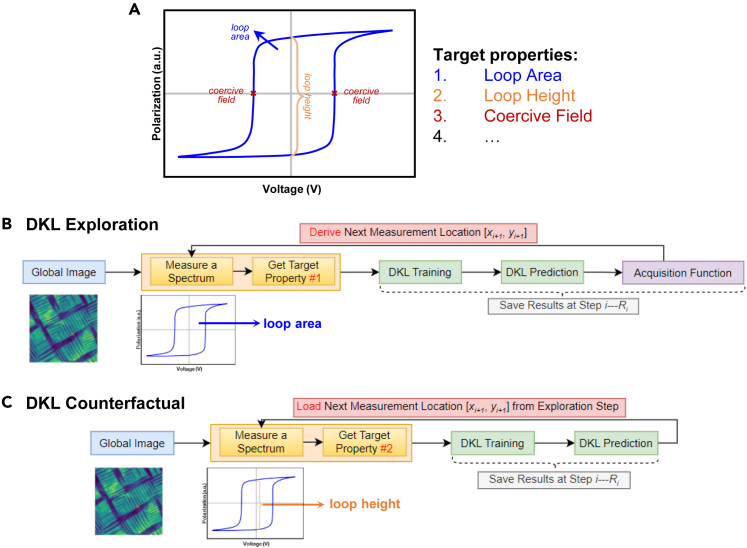
Counterfactual analysis (A–C) Workflow of counterfactual analysis. (A) There are multiple target properties encoded in spectroscopic data. (B) DKL exploration based on one target property determines the measurement location at each step. (C) In counterfactual analysis, another target property will be used to perform DKL analysis, and the measurement location at each step is loaded from the corresponding exploration step. (D) DKL counterfactual analysis results, where the DKL exploration is guided by the target property of loop area, and counterfactual analysis target properties are loop height and coercive field. DKL analysis based on random location is also plotted as a comparison.

**Figure 6:**
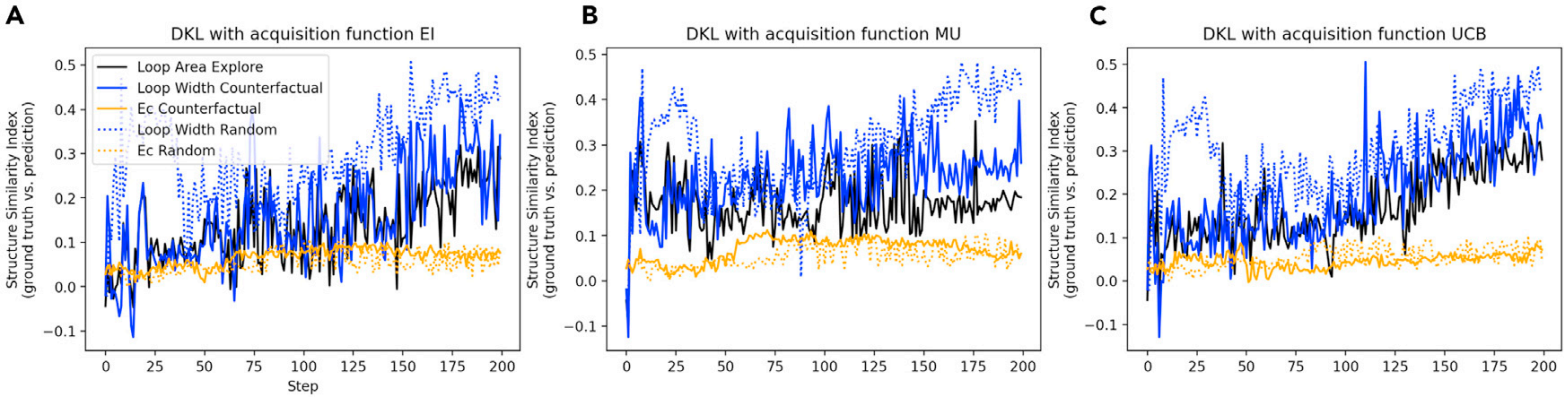
Counterfactual analysis results, where the DKL exploration is guided by the target property of loop area, and counterfactual analysis target properties are loop height and coercive field; DKL analysis based on random location plotted as a comparison (A–C) Shown are the counterfactual analysis results of DKL with different acquisition functions expected improvement (EI), maximum uncertainty (MU), and upper confidence bound (UCB), respectively. The DKL counterfactual analysis process is shown in Figure 5.

This counterfactual analysis allows us to determine whether the sampled points based on target property 1 help explore target property 2. To illustrate this concept, we have performed DKL exploration with loop area from polarization-voltage hysteresis, as shown in Figure 5B. This creates an *experimental trace*, meaning the sequence of the sampling points, corresponding image patches, and hysteresis loops. With the experimental trace for loop area as a scalarizer created, we have loaded the sampling points and used loop height or coercive field to perform DKL analysis, as shown in Figure 5C. In addition to DKL counterfactual analysis that uses DKL sampling points based on a different target property, we also performed DKL analysis using random sampling points as a comparison. DKL counterfactual and random analysis results are shown in Figure 6.

As a measure of the counterfactual experiment progression, we compare the spatial distribution of the predicted images with the ground truth. Here, we generate the DKL prediction image of target functionality and calculate the structural similarity index (SSID) between the DKL prediction and ground truth. We expect that, if the physical behaviors described by different scalarizers are strongly correlated, the SSID values will be high, whereas if they differ, the SSID will be low.

Shown in Figure 6 are the DKL counterfactual analysis results with different acquisition functions. The experimental trace is created by performing DKL exploration with the polarization-voltage hysteresis loop area as the target property. Then, we loaded the experimental trace and used loop height and coercive field to perform counterfactual analysis. The SSID evolution of DKL exploration (with loop area as target property) and DKL counterfactual with loop height as target property are similar, as shown in Figure 6. However, the evolution of DKL counterfactual with the coercive field as target property is slightly different from the DKL exploration. This is most likely because the loop area and loop height originate from similar physical mechanisms, i.e., remnant polarization. In contrast, the coercive field is tied to different physical mechanisms.

We further proceed to define *trajectory analysis* and *feature discovery*. We note that AE in physical imaging traces a certain trajectory in the image plane of the system. Given that the global image is available before the DKL experiment, this trajectory can be visualized and examined in real space both in real time and after the experiment.

**Figure 7:**
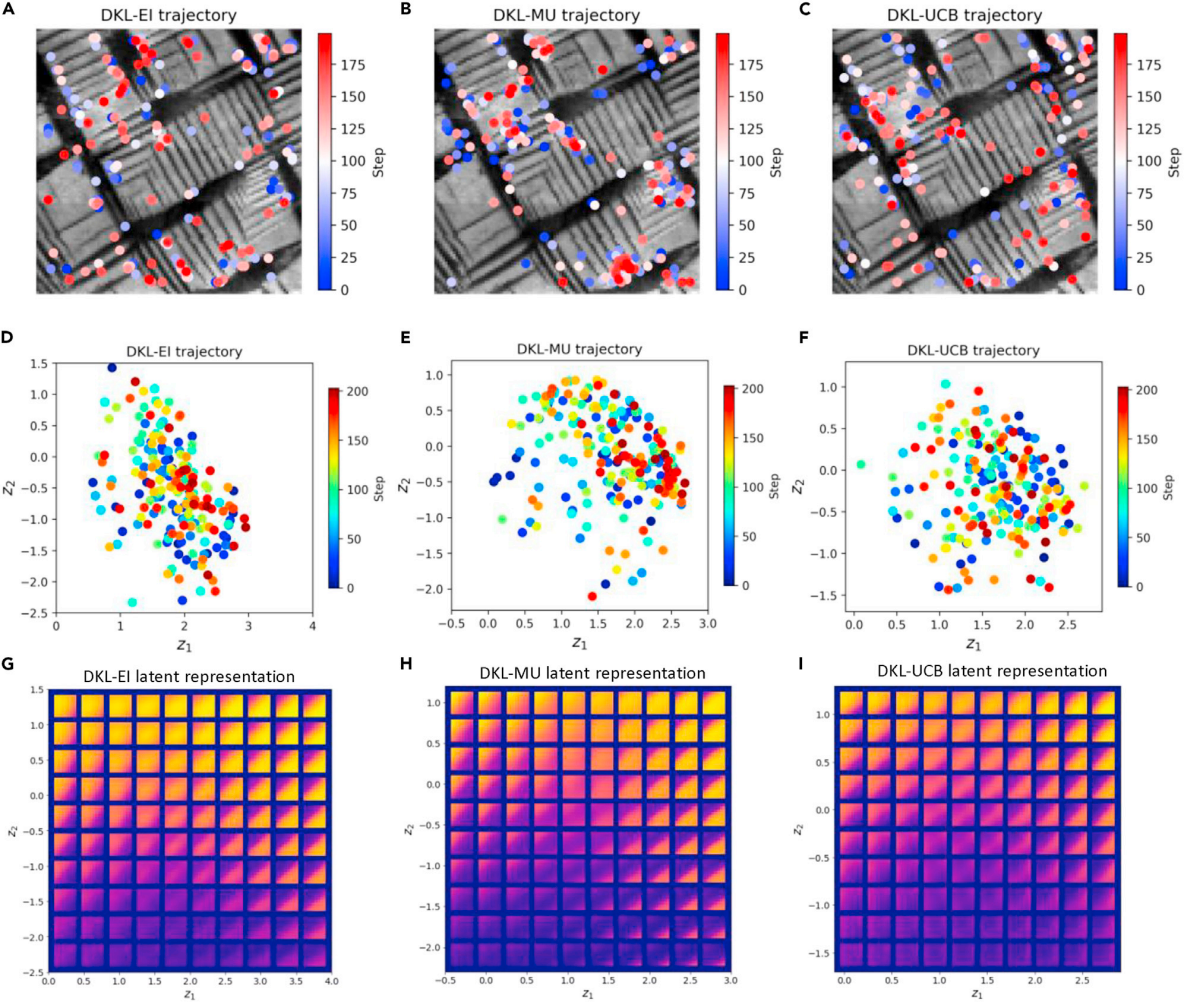
Trajectories of DKL exploration with different acquisition functions and evolution of latent component *Z*_*1*_, *Z*_*2*_ as a function of step (A–C) In the trajectory of DKL exploration with different acquisition functions EI, MU, and UCB, respectively, where the exploration locations are shown on the structural image, the spot’s color represents a step. (D–F) Trajectory in latent space, where (D), (E), and (F) correspond to the trajectories of DKL exploration with difference acquition functions EI, MU, and UCB, respectively. (G–I) rVAE latent representations, where the rVAE is trained with DKL samplings with different acquition fucntions EI, MU, and UCB, respectively.

Shown in Figures 7A–7C are the trajectories of the DKL exploration. The exploration locations are plotted in the structural image in sequence from blue to red. The examination of the real-space trajectory yields a powerful real-time and post-experimental analysis tool to monitor the progression of the AE. For example, the concentration of the experimental points in a certain part of the image plane to full exclusion of other regions often suggests the effects of instrumental crosstalk (e.g., tilt). Secondly, visual examination of the trajectory vs. the structural image allows direct identification of the microstructural elements that carry functionalities of interest discovered by the DKL. For example, in Figures 7A and 7C, many of the experimental points have been chosen at the ferroelastic domain boundaries between large *a* and *c* domains. At the same time, the sampling of the small *a-c* domain stripes is very sparse, suggesting that the functionality of interest (i.e., hysteresis loop area) does not manifest strongly in these regions even though they comprise most of the sample surface. Note that this analysis can be further extended toward human-in-the-loop analysis, where supervised ML can be used to identify objects of interest (human-provided goals and labels), and the next round of the AE will be focused only on these specific features. This approach has been demonstrated for the analysis of grain boundaries in hybrid perovskites.^43^

During the automated experiment, the ML agent learns which image patches correspond to the optimization of the scalarizer function. To explore the dynamics of this learning process and explore what are the discovered features, we introduce the latent analysis with rotationally invariant variational autoencoders (rVAEs) on the full experimental trace.^44^^,^^45^^,^^46^ The rVAE disentangles the factors of variation in all image patches into latent variables; in this case, the latent space shows the structural variations that are somehow relevant to physical features (e.g., domains). Shown in Figures 7D–7F are the trajectories of DKL exploration in rVAE latent space. The corresponding latent representations are shown in Figures 7G–7I and allow us to identify the physical features related to latent variables. Note that the analysis above is based on the data contained in the experimental trace and becomes available after the experiment.

Finally, to explore the discovery process, we introduce *latent trajectory analysis*. In this case, we utilize the fact that the global image and hence full collection of the image patches are available before the experiment. This allows the latent space of the system to be constructed via the suitable (invariant) VAE. The image patches that become available as the experimental trace can be visualized in the corresponding latent space, whereas the trajectory in the global latent space and the evolution of latent variables along the experimental path can be visualized in real time. Note that these latent variables are different from the DKL latent, since the DKL embeddings are updated at each experimental step (and their meaning can be determined only via backpropagation through the deep convoltional neural networks).

**Figure 8:**
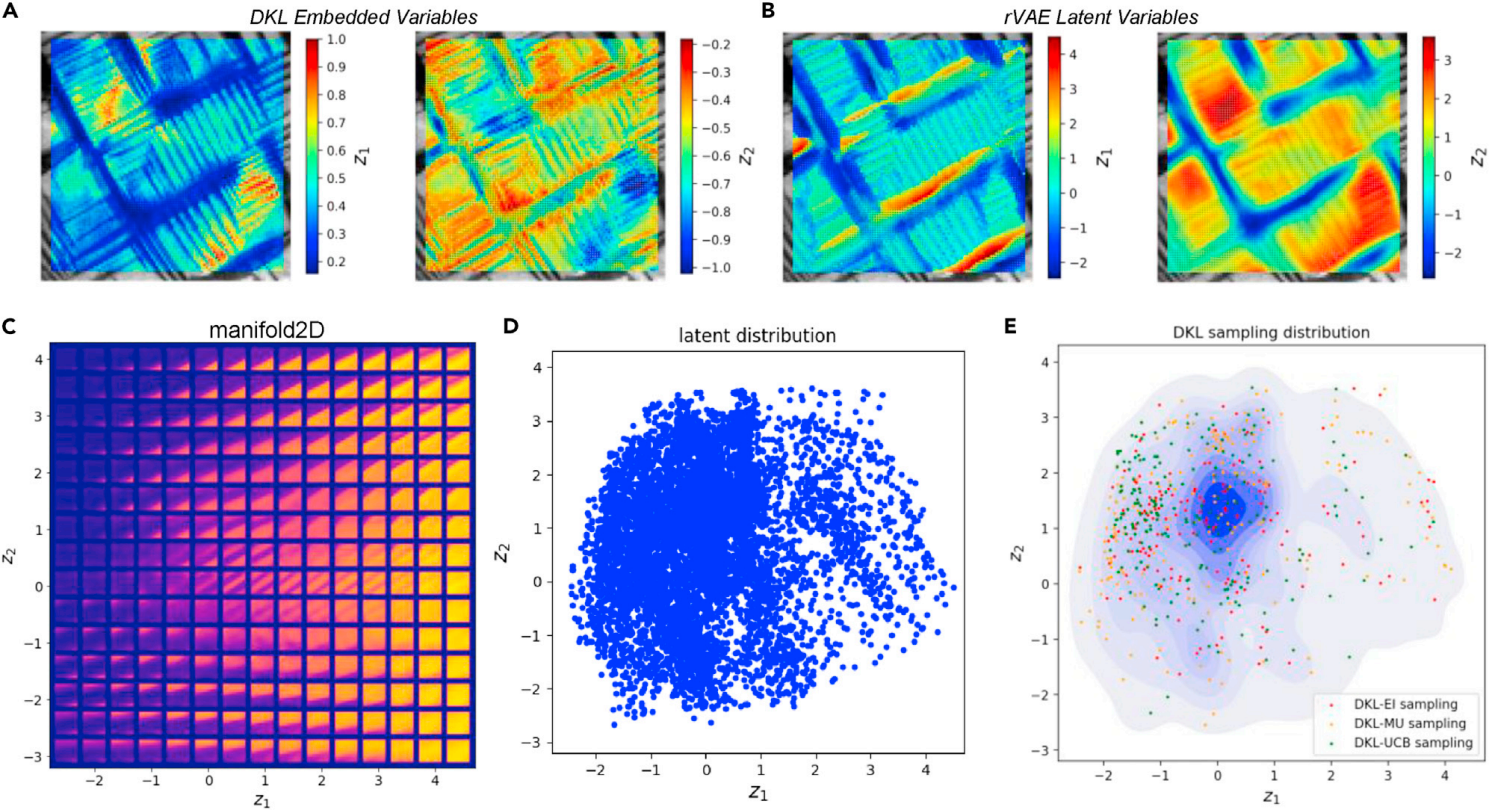
rVAE analysis of DKL sampling (A) DKL embedded variables. (B) rVAE latent variables. (C and D) rVAE analysis of all image patches and the DKL sampling distribution. Here, (D) is the latent distribution of the system, i.e., all image patches represented in the latent space. (E) Shown is the superposition of the sampled patches on the kernel density estimate of the full latent distribution of the system, showing which of the regions were sampled for different acquisition functions. These data can be color-coded by the number of points (not shown).

The latent trajectory analysis is shown in Figures 8A and 8B. Figure 8A shows the correlation of DKL embedded variables and structural factors, and Figure 8B shows the correlation between rVAE latent variables and structural factors, where there is some similarity between DKL and rVAE variables. Figures 8C and 8D indicate the distribution of DKL samplings in the rVAE latent space. Note that for all three acquisition functions, the AE samples the regions in the latent space corresponding to *a-c* domain walls; from the point of view of ferroelectric physics, this can be understood that more factors (wall, strain, defect, etc.) affect properties near *a-c* domain walls. The central peak of the kernel density estimate corresponding to most usual microstructures (dense ferroelastic domain patterns) remains unaddressed. This approach illustrates which microstructures give rise to the thought behaviors. Note that additional insight into this discovery process can be derived by plotting the time dependence of the latent variables, time-coding trajectories for a single acquisition function in the latent space, etc.

Here we want to note that the structural factors can be modified by changing the image patch size, and correspondingly, the DKL exploration trajectory and samplings will also be changed. We believe this change is tied to the physics involved in the image patches with different sizes. Note that the discovery process can be further explored by exploring the time dependence of the latent codes corresponding to the patches along the experimental trace.

## Discussion

The AE post-experimental analysis represented above illustrates the collection of descriptors available during and after the DKL automated experiment that provide insight into the progression of the training (predictive uncertainties), rate of the BO of target functionalities, and real-space and latent space discovery trajectories. Given the rich nature of the information contained in the experimental trace, these methodologies can be developed further using multiple tools developed in the context of static ML and BO. For example, the analysis can be extended to exploring the emergence of correlations between image and spectral data in trace via linear (canonical correlation analysis)^47^ and VAE-based methods. The patches in trace or spectra can be used as labels for the semi-supervised analysis of the global structural data. Multiple opportunities further emerge for the AE policies, including the introduction of multi-objective optimization for multiple scalarizers, changing policies during the experiment, etc. We defer the analysis of these opportunities for further experimental effort.

**Figure 9:**
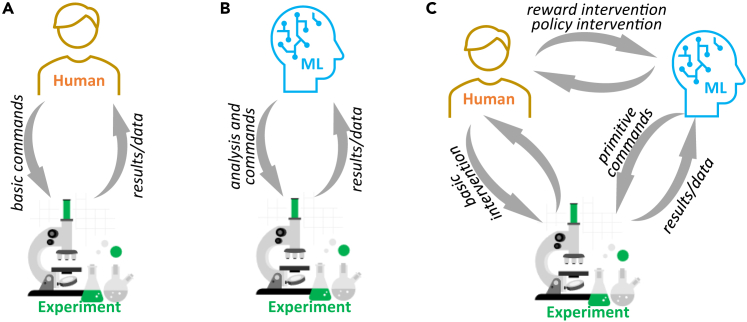
Human- vs. artificial intelligence-assisted experiments (A) In the human-controlled paradigm, the human operator issues control commands in the instrument-specific hyper-language for certain experiments. (B) In ML-driven autonomous experiments, the human operator is substituted by the AI agent. (C) In the AI-assisted human-in-the-loop paradigm, the human operator oversees experiments run by the AI agent. During this, humans can directly intervene in the experiment or tune (in real time) the decision-making process of the ML agent, which requires developing the methodology to monitor and intervene in the ML-driven autonomous experiments.

Finally, we consider the opportunities for human-in-the-loop interventions in the automated experiment, as shown in Figure 9. Table 1 summarizes the definitions for the real-time and post-experimental analysis descriptors in the AE. The indicators such as learning and regret curves and real-space and latent trajectories can be visualized in real time during the experiment and provide strong signals on the progress in predictability and discovery during AE and the nature of uncovered structural elements and functional behaviors. At the same time, AE allows easy access to the control parameters. The BO pathways can be tuned via the selection of the scalarizer function that can be chosen from the pre-populated list or dynamically tuned during the experiment (e.g., signal averaged over selected spectral band). The balance between exploration and exploitation can be tuned via the acquisition function, again selecting from the list or tuning the weight coefficients in UCB, etc. Similarly, random exploration can be added via the epsilon-greedy term.

**Table 1:** Summary of the definitions for AE post-experimental analysis descriptors

Characteristic	Definition	Availability
Global image	initial structural dataset available before DKL experiment; used to create patches for DKL training	before
DKL latent	the latent variables encoding the structural information in the patches	during^b^
Scalarizer function	the function defining what characteristic of the spectrum guides Bayesian optimization	before^a^
Acquisition function	function combining DKL prediction and uncertainty of the scalarizer function	before^a^
Policy	principle for selection of next path; the simplest policy is maximization of acquisition function but can be more complex including epsilon-greedy or switching between multiple scalarizers or acquisition functions; human-in-the-loop intervention tunes some aspects of the policy	before^a^
Experimental trace	collection of patches (and their coordinates) and spectra derived during the experiment; trace and global image are the results of AE SPM	during
Live DKL model	DKL model in the state corresponding to the *n*-th experimental step	during
Final DKL model	DKL model in the state corresponding to the end of the experiment	after
Complete DKL model	DKL model trained on the full dataset (if available from grid measurements, etc.)	not available in real experiments
Regret analysis	the difference between predictions of the live DKL model and the final DKL model after the whole experiment (i.e., after 200 steps in this work)	during^b^ and after
Learning curve	change of the DKL uncertainty (mean and deviation), indicative of the predictability of the patch-scalarizer relationship	during
Counterfactual scalarizer	the availability of full spectral data as a part of the experimental trace allows us to estimate what the BO step would be if the scalarizer were chosen to be different	during
Trajectory analysis	real-time trajectory of the probe that can be represented in the global image plane	during
Feature discovery	analysis of the latent variables and latent representations of image patches and spectra in the trace; here, we realize only patch analysis, but extension to spectra is straightforward	after
Latent trajectory analysis	analysis of the experimental trajectory in the latent space of the full collection of the image patches derived from the global image	during^b^

a Denotes parameters that provide controls for human-in-the-loop intervention.

b Denotes observables that can be naturally monitored during the DKL experiment to make human-in-the-loop decisions. Note that strictly speaking all after-experiment descriptors can be evaluated on the fly, but they represent more difficult to interpret and intervene upon behaviors.

Several of the parameters (e.g., related to the final DKL model or feature discovery) are available at the end of the DKL experiment. Rigorously, these can be updated throughout the experiment. However, we believe that their interpretability makes them ideal for the human-in-the-loop interventions, where the experiment is paused, and the human operator tunes the experimental policies. We also note that this analysis can be further extended to introduce additional knowledge during the experiment, for example, by using a deep convolutional network to perform the image segmentation and run DKL on segmented (rather than raw) data. We hope that the provided notebooks^48^ will allow the broad experimental community to explore these opportunities.

To summarize, here we proposed and implemented the AE post-experimental analysis for the automated experiment in SPM. This analysis is implemented on a pre-acquired dataset, thus allowing a comparison of the dissimilar experimental policies. However, it can be implemented on any microscope equipped with previously reported DKL workflows. Overall, this analysis allows monitoring the progression of the exploratory and exploitative descriptors during the AE and introduces the strategies for human-in-the-loop intervention based on the target and policy controls.

The proposed approach can be extended to all other imaging spectroscopic methods, including STEM-EELS, 4D STEM, optical microscopy, and scanning electron microscopy combined with nanoindentation and multiple chemical imaging methods. The only requirement for the applicability of AE post-experimental analysis in its present form is the availability of the structure-spectra pairs as the basis for the imaging process. From the instrumental side, the common denominator is the control hyper-language that gives access to the probe position and enables the initiation of imaging and spectroscopic scans.

We further note that this methodology can be applied to more complex scenarios, including exploration of the parameter space of the theoretical models or composition and processing spaces in automated materials synthesis, as well as chemical spaces for organic molecules or biomolecules. However, in these cases, the measures for exploration and representation will depend on the structure and correlations in the corresponding parameter spaces, necessitating the development of domain-specific descriptors. Overall, we believe that the proposed framework opens the pathway to interpretable automated experiments, AE monitoring, and human-in-the-loop interventions.

## Experimental procedures

### Resource availability

#### Lead contact

Requests for information and resources used in this article should be addressed to the lead contact Yongtao Liu (liuy3@ornl.gov).

#### Materials availability

There is no new material generated from this work.

## Data and code availability

The analysis codes that support the findings of this study are available at https://github.com/yongtaoliu/AE-PostExperimentAnalysis-DKL-BEPS and Zenodo https://doi.org/10.5281/zenodo.8002277.^48^

This analysis uses AtomAI package at https://github.com/pycroscopy/atomai ^49^^,^^50^ and pyroVED package at https://github.com/ziatdinovmax/pyroVED.^51^

## References

[1] Higgins K., Valleti S.M., Ziatdinov M., Kalinin S.V., Ahmadi M. (2020). Chemical robotics enabled exploration of stability in multicomponent lead halide perovskites via machine learning. ACS Energy Lett..

[2] Higgins K., Ziatdinov M., Kalinin S.V., Ahmadi M. (2021). High-throughput study of antisolvents on the stability of multicomponent metal halide perovskites through robotics-based synthesis and machine learning approaches. J. Am. Chem. Soc..

[3] Bennett J.A., Abolhasani M. (2022). Autonomous chemical science and engineering enabled by self-driving laboratories. Current Opinion in Chemical Engineering.

[4] Epps R.W., Volk A.A., Ibrahim M.Y., Abolhasani M. (2021). Universal self-driving laboratory for accelerated discovery of materials and molecules. Chem.

[5] Seifrid M., Pollice R., Aguilar-Granda A., Morgan Chan Z., Hotta K., Ser C.T., Vestfrid J., Wu T.C., Aspuru-Guzik A. (2022). Autonomous chemical experiments: Challenges and perspectives on establishing a self-driving lab. Acc. Chem. Res..

[6] Epps R.W., Volk A.A., Reyes K.G., Abolhasani M. (2021). Accelerated AI development for autonomous materials synthesis in flow. Chem. Sci..

[7] Reis M., Gusev F., Taylor N.G., Chung S.H., Verber M.D., Lee Y.Z., Isayev O., Leibfarth F.A. (2021). Machine-Learning-Guided Discovery of 19F MRI Agents Enabled by Automated Copolymer Synthesis. J. Am. Chem. Soc..

[8] Ahmadi M., Ziatdinov M., Zhou Y., Lass E.A., Kalinin S.V. (2021). Machine learning for high-throughput experimental exploration of metal halide perovskites. Joule.

[9] Ren F., Ding X., Zheng M., Korzinkin M., Cai X., Zhu W., Mantsyzov A., Aliper A., Aladinskiy V., Cao Z. (2023). AlphaFold Accelerates Artificial Intelligence Powered Drug Discovery: Efficient Discovery of a Novel CDK20 Small Molecule Inhibitor. Chem. Sci..

[10] Gongora A.E., Xu B., Perry W., Okoye C., Riley P., Reyes K.G., Morgan E.F., Brown K.A. (2020). A Bayesian experimental autonomous researcher for mechanical design. Sci. Adv..

[11] Ferguson A.L., Brown K.A. (2022). Data-driven design and autonomous experimentation in soft and biological materials engineering. Annu. Rev. Chem. Biomol. Eng..

[12] Roccapriore K.M., Boebinger M.G., Dyck O., Ghosh A., Unocic R.R., Kalinin S.V., Ziatdinov M. (2022). Probing Electron Beam Induced Transformations on a Single-Defect Level via Automated Scanning Transmission Electron Microscopy. ACS Nano.

[13] Roccapriore K.M., Dyck O., Oxley M.P., Ziatdinov M., Kalinin S.V. (2022). Automated experiment in 4D-STEM: exploring emergent physics and structural behaviors. ACS Nano.

[14] Roccapriore K.M., Kalinin S.V., Ziatdinov M. (2022). Physics discovery in nanoplasmonic systems via autonomous experiments in scanning transmission electron microscopy. Adv. Sci..

[15] Kalinin S.V., Vasudevan R., Liu Y., Ghosh A., Roccapriore K., Ziatdinov M. (2023). Probe microscopy is all you need. Mach. Learn, Sci. Technol..

[16] Rashidi M., Wolkow R.A. (2018). Autonomous scanning probe microscopy in situ tip conditioning through machine learning. ACS Nano.

[17] Liu Y., Morozovska A.N., Eliseev E.A., Kelley K.P., Vasudevan R., Ziatdinov M., Kalinin S.V. (2023). Autonomous scanning probe microscopy with hypothesis learning: Exploring the physics of domain switching in ferroelectric materials. Patterns.

[18] Thomas J.C., Rossi A., Smalley D., Francaviglia L., Yu Z., Zhang T., Kumari S., Robinson J.A., Terrones M., Ishigami M. (2022). Autonomous scanning probe microscopy investigations over WS2 and Au {111. npj Comput. Mater..

[19] Vasudevan R.K., Kelley K.P., Hinkle J., Funakubo H., Jesse S., Kalinin S.V., Ziatdinov M. (2021). Autonomous experiments in scanning probe microscopy and spectroscopy: choosing where to explore polarization dynamics in ferroelectrics. ACS Nano.

[20] Liu Y., Yang J., Vasudevan R.K., Kelley K.P., Ziatdinov M., Kalinin S.V., Ahmadi M. (2023). Exploring the Relationship of Microstructure and Conductivity in Metal Halide Perovskites via Active Learning-Driven Automated Scanning Probe Microscopy. J. Phys. Chem. Lett..

[21] Krull A., Hirsch P., Rother C., Schiffrin A., Krull C. (2020). Artificial-intelligence-driven scanning probe microscopy. Commun. Phys..

[22] Gordon O.M., Moriarty P.J. (2020). Machine learning at the (sub) atomic scale: next generation scanning probe microscopy. Mach. Learn, Sci. Technol..

[23] Liu Y., Kelley K.P., Vasudevan R.K., Zhu W., Hayden J., Maria J.P., Funakubo H., Ziatdinov M.A., Trolier-McKinstry S., Kalinin S.V. (2022). Automated experiments of local non-linear behavior in ferroelectric materials. Small.

[24] Liu Y., Kelley K.P., Funakubo H., Kalinin S.V., Ziatdinov M. (2022). Exploring physics of ferroelectric domain walls in real time: deep learning enabled scanning probe microscopy. Adv. Sci..

[25] Ziatdinov M., Liu Y., Kelley K., Vasudevan R., Kalinin S.V. (2022). Bayesian active learning for scanning probe microscopy: from gaussian processes to hypothesis learning. ACS Nano.

[26] McDannald A., Frontzek M., Savici A.T., Doucet M., Rodriguez E.E., Meuse K., Opsahl-Ong J., Samarov D., Takeuchi I., Ratcliff W., Kusne A.G. (2022). On-the-fly autonomous control of neutron diffraction via physics-informed Bayesian active learning. Appl. Phys. Rev..

[27] Noack M.M., Zwart P.H., Ushizima D.M., Fukuto M., Yager K.G., Elbert K.C., Murray C.B., Stein A., Doerk G.S., Tsai E.H.R. (2021). Gaussian processes for autonomous data acquisition at large-scale synchrotron and neutron facilities. Nat. Rev. Phys..

[28] Maruyama S., Ouchi K., Koganezawa T., Matsumoto Y. (2020). High-Throughput and Autonomous Grazing Incidence X-ray Diffraction Mapping of Organic Combinatorial Thin-Film Library Driven by Machine Learning. ACS Comb. Sci..

[29] Casukhela R., Vijayan S., Jinschek J.R., Niezgoda S.R. (2022). A Framework for the Optimal Selection of High-Throughput Data Collection Workflows by Autonomous Experimentation Systems. Integr. Mater. Manuf. Innov..

[30] Kalinin S.V. (2023). Designing Workflows for Materials Characterization. arXiv.

[31] Liu Y., Kelley K.P., Vasudevan R.K., Funakubo H., Ziatdinov M.A., Kalinin S.V. (2022). Experimental discovery of structure–property relationships in ferroelectric materials via active learning. Nat. Mach. Intell..

[32] Yager K.G., Majewski P.W., Noack M.M., Fukuto M. (2023). Autonomous x-ray scattering. Nanotechnology.

[33] Lei B., Kirk T.Q., Bhattacharya A., Pati D., Qian X., Arroyave R., Mallick B.K. (2021). Bayesian optimization with adaptive surrogate models for automated experimental design. npj Comput. Mater..

[34] Ziatdinov M.A., Liu Y., Morozovska A.N., Eliseev E.A., Zhang X., Takeuchi I., Kalinin S.V. (2022). Hypothesis learning in automated experiment: application to combinatorial materials libraries. Adv. Mater..

[35] Bateni F., Epps R.W., Antami K., Dargis R., Bennett J.A., Reyes K.G., Abolhasani M. (2022). Autonomous nanocrystal doping by self-driving fluidic micro-processors. Advanced Intelligent Systems.

[36] Garnett R. (2023).

[37] Martin O. (2018).

[38] Liu Y., Zhong Y., Zheng B., Liu Y. (2023). Learning the right channel in multimodal imaging: automated experiment in Piezoresponse Force Microscopy. Prog. Orthod..

[39] Jesse S., Vasudevan R.K., Collins L., Strelcov E., Okatan M.B., Belianinov A., Baddorf A.P., Proksch R., Kalinin S.V. (2014). Band Excitation in Scanning Probe Microscopy: Recognition and Functional Imaging. Annu. Rev. Phys. Chem..

[40] Liu Y., Ziatdinov M., Kalinin S.V. (2022). Exploring causal physical mechanisms via non-gaussian linear models and deep kernel learning: applications for ferroelectric domain structures. ACS Nano.

[41] Shahriari B., Swersky K., Wang Z., Adams R.P., De Freitas N. (2016). Taking the human out of the loop: A review of Bayesian optimization. Proc. IEEE.

[42] Roccapriore K.M., Dyck O., Oxley M.P., Ziatdinov M., Kalinin S.V. (2022). Automated experiment in 4D-STEM: exploring emergent physics and structural behaviors. ACS Nano.

[43] Liu Y. (2022). Exploring the microstructural origins of conductivity and hysteresis in metal halide perovskites via active learning driven automated scanning probe microscopy. arXiv.

[44] Liu Y., Proksch R., Wong C.Y., Ziatdinov M., Kalinin S.V. (2021). Disentangling ferroelectric wall dynamics and identification of pinning mechanisms via deep learning. Adv. Mater..

[45] Kalinin S.V., Steffes J.J., Liu Y., Huey B.D., Ziatdinov M. (2021). Disentangling ferroelectric domain wall geometries and pathways in dynamic piezoresponse force microscopy via unsupervised machine learning. Nanotechnology.

[46] Kalinin S.V., Dyck O., Ghosh A., Liu Y., Sumpter B.G., Ziatdinov M. (2023). Unsupervised machine learning discovery of structural units and transformation pathways from imaging data. APL Machine Learning.

[47] Ziatdinov M., Fujii S., Kiguchi M., Enoki T., Jesse S., Kalinin S.V. (2016). Data mining graphene: correlative analysis of structure and electronic degrees of freedom in graphenic monolayers with defects. Nanotechnology.

[48] Liu Y. (2023). Forensics-DKL-BEPS. Zenodo.

[49] Ziatdinov M.A. (2023). AtomAI. https://github.com/pycroscopy/atomai.

[50] Ziatdinov M., Ghosh A., Wong C.Y., Kalinin S.V. (2022). AtomAI framework for deep learning analysis of image and spectroscopy data in electron and scanning probe microscopy. Nat. Mach. Intell..

[51] Ziatdinov M.A. (2023). pyroVED. https://github.com/ziatdinovmax/pyroVED.

